# Reconstructing genetic mating systems in the absence of parental information in colonially breeding waterbirds

**DOI:** 10.1186/1471-2148-11-196

**Published:** 2011-07-08

**Authors:** Carolina I Miño, Michael A Russello, Priscila F Mussi Gonçalves, Silvia N Del Lama

**Affiliations:** 1 Laboratório de Genética de Aves, Departamento de Genética e Evolução, Universidade Federal de São Carlos, São Carlos, 13565-905, São Paulo, Brazil; 2 Department of Biology, The University of British Columbia, Okanagan Campus, 3333 University Way, Kelowna, V1V 1V7, British Columbia, Canada

## Abstract

**Background:**

DNA-based studies have demonstrated that avian genetic mating systems vary widely, with many species deviating from long-assumed monogamy by practicing extra-pair paternity and conspecific brood parasitism. Colonially breeding waterbirds provide interesting models in which to investigate this question because they show nesting habits proposed to promote alternative reproductive strategies. However, little is known about the genetic mating systems of this group of birds, mainly due to difficulties in obtaining genetic data from incubating adults at nests that are necessary for conducting conventional parentage studies. Here, we inferred kinship patterns among offspring in broods of three co-distributed waterbird species, Wood Stork (*Mycteria americana*), Roseate Spoonbill (*Platalea ajaja*) and Great Egret (*Ardea alba egretta*), to investigate genetic mating system in the absence of parental data.

**Results:**

Multi-step analyses combining estimates of relatedness coefficients, formulation of relationship-hypotheses, significance testing of alternative hypotheses, and maximum-likelihood sibship reconstruction techniques revealed evidence that alternative reproductive strategies may be present in natural populations of Wood Storks and Roseate Spoonbills, whereas relatedness of co-nestlings diagnosed in the Great Egrets did not deviate from a hypothesis of genetic monogamy. Specifically, under this analytical framework, inferred kinship relationships revealed that Great Egret nests contained full-sibling nestlings (100%), with the Roseate Spoonbill (RS) and Wood Stork (WS) exhibiting proportions of half-siblings (RS: 5%) and/or unrelated nestlings (RS: 24%; WS: 70%), patterns consistent with extra-pair paternity and conspecific brood parasitism, respectively.

**Conclusions:**

We provide evidence that genetic monogamy occurs in Brazilian natural breeding colonies of the Great Egret, but is not the sole reproductive strategy employed by the Wood Stork and the Roseate Spoonbill. In fact, extra-pair paternity and conspecific brood parasitism were common in the latter two species, with a combined frequency of 7.5% and 11.3% in Roseate Spoonbill and Wood Stork, respectively. Although geographically co-distributed, differences among these species may be due to variation in their life histories. From a methodological standpoint, the approach implemented here, although not free from limitations, can have broad application for analyzing systems with limited genealogical information and/or in studying similarly challenging organisms in which obtaining genetic data on complete families is problematic.

## Background

Avian mating systems vary substantially among and within species, with DNA-based studies revealing that genetic mating system frequently deviates from the social monogamy observed in many bird species [1]. Genetic mating systems have been traditionally characterized by means of DNA-based parentage assignments, comparing the genotypes of caretaker adults to those of putative offspring within a brood [2]. In many species, however, obtaining an appropriate sample of candidate parents is not straightforward or possible in natural populations. For example, birds such as parrots, grouse, birds of prey and waterbirds are elusive animals, difficult to capture, often inhabit inaccessible or remote places, and can move long distances, imposing difficulties to direct blood sampling of putative parents [3-5]. Kinship analyses made possible by use of hypervariable markers such as microsatellites have greatly facilitated the study of avian mating systems [1,6,7]. Even in the absence of biological samples from putative parents, kinship reconstruction techniques may provide insights into relative relatedness and mating system when putative groups of siblings can be determined *a priori *[2], as is the case for all nestlings within a brood.

Waterbirds of the order Ciconiiformes and Pelecaniiformes are colonial breeders that prove interesting models in which to investigate alternative reproductive tactics. In general, colonially breeding species are predicted to have high extra-pair paternity (EPP) rates due to the close proximity to potential sexual partners [8]. Extra-pair copulation (EPC), i. e. copulation between one individual and another of the opposite sex that is not its social partner, a pre-requisite for EPP, can be frequent in breeding colonies [9-11]. High densities of reproductive individuals clustered together are, in turn, one of the most important ecological factors affecting EPP at the species level [11]. Colonial nesters also can be prone to conspecific brood parasitism (CBP), due to the availability, within colonies, of potential hosts' nests in high numbers [12-15]. In spite of showing an interesting breeding behavior, the genetic mating systems of colonial waterbirds, particularly those of Neotropical species, are not well studied [16].

One reason for the dearth of studies of genetic mating systems in waterbirds is that many species exhibit adult behaviour and life-history characteristics that preclude the ascertainment of biological samples for DNA analysis. For example, breeding adults of some storks, spoonbills and egrets show restless behaviour and fly away from their nests as soon as researchers enter the breeding colonies to conduct fieldwork, preventing their capture and hindering the collection of blood samples for DNA analyses. Although non-invasive methods have been applied in some avian species for the collection of parental genetic material, those methods may not be suitable for the study of natural breeding colonies of waterbirds. Egg-swabbing, for instance, requires relatively extensive early-egg manipulation [17]. This approach will increase the amount of time spent by researchers in the breeding colonies in this critical precocious stage of development, and consequently will increase nest disturbance and augment the risk of nest loss. Likewise, some waterbirds' breeding colonies, such as those located in the Pantanal wetland of Brazil, have a high incidence of aerial predators (e.g., the Southern Caracara, Turkey Vulture and Black-collared Hawk) [18]. Presence of researchers in and around nests often induces adults to fly away, offering predators enhanced opportunities to assault eggs. The use of blood sucking insects is another non-invasive technique that has been successfully applied to sample material of incubating birds [19]. This methodology requires checking dummy eggs every ca. 30 min to see if the insects have had their blood meal and to collect blood [19]. Storks, herons and spoonbills nests are built in trees as tall as 20 m (e.g., in breeding colonies from the Brazilian Pantanal and Amapa state), which can be accessed only by professional tree-climbers. Thus, direct application of non-invasive sampling techniques is precluded in such situations.

In this study, we inferred kinship patterns among offspring in broods of three geographically co-distributed species, Wood Stork (*Mycteria americana *Linnaeus, 1758), Roseate Spoonbill (*Platalea ajaja *Linnaeus, 1758) and Great Egret (*Ardea alba egretta*, Gmelin 1789), to investigate genetic mating system in the absence of parental data. Our prediction was that if social mating system (monogamy) equates to genetic mating system in these species, all nestlings found inside a nest will be genetic full-siblings. We combined different approaches based on multilocus microsatellite genotypes to reconstruct kinship among nestlings: estimates of relatedness coefficients, formulation of relationship-hypotheses, significance testing of alternative hypotheses, and maximum-likelihood sibship reconstruction techniques. Using this framework, we found evidence that alternative reproductive strategies may be present in natural populations of Wood Storks and Roseate Spoonbills, whereas Great Egrets did not deviate from a hypothesis of genetic monogamy. Although co-distributed, differences among these species may be due to variation in their life histories. In addition, we explored the relative merits and drawbacks of our approach for reconstructing genetic mating system in the absence of parental information, including its applicability to future studies.

## Methods

### Study species

The Wood Stork occurs from the southeastern United States of America (U.S.A.) to northern Argentina [20]. Breeding colonies of this species can be found in major Brazilian wetlands, from the states of Para and Amapa in the north of the country to the Pantanal region in the center-west (which includes the states of Mato Grosso and Mato Grosso do Sul) [21]. This species lays one brood per season, with a time interval of up to two days between eggs; the standard size clutch is three eggs [22]. Limited behavioural observations have described the Wood Stork as socially monogamous, with both parents sharing incubation (27 to 32 d.) and nestling care [22]. Nestlings are classified as altricial. At least one parent attends the nest full time until the chicks are 20 days old; nest attendance declines progressively with time, but the young return to the nest to be fed until they are 80 days old [23].

The Roseate Spoonbill breeds from southeastern U.S.A. to central Argentina [24]. In Brazil, colonies can be found along the equatorial line in the state of Amapa [25], in the center-west of the country in the Pantanal region [21], and in the wetlands of Rio Grande do Sul state, in the south of the country [26,27]. Standard clutch size in this species is three eggs (range 1-5) [24]. Roseate Spoonbills have long been considered socially monogamous based on limited behavioural observations of one pair during one reproductive cycle [24]. Both parents share incubation (22 d.) and nestling care. Nestlings are semi-altricial and depend on parents for feeding until ca. 67 days old [24].

The Great Egret breeds from southern Canada and U.S.A. to Chile and Argentina [28]. Breeding colonies of this species can be found in the north, center-west, south and southwest of Brazil [29]. This waterbird is socially monogamous [30], but extra-pair copulation (EPC) has been reported with varying frequency [9,31,32]. Standard clutch size in this species is three eggs; both parents incubate eggs (23-27 days) taking turns every 24 h [33]. Nestlings are classified as semi-altricial. Both adults feed nestlings during the first week of age, taking turns in nestling care during the following weeks [33]. When nestlings are 21-30 days old they begin to move away from nests, progressively extending the time they spend away from them, and abandoning nests altogether at 62 days old [33].

### Sampling, DNA isolation and genotypic data collection

**Figure 1:**
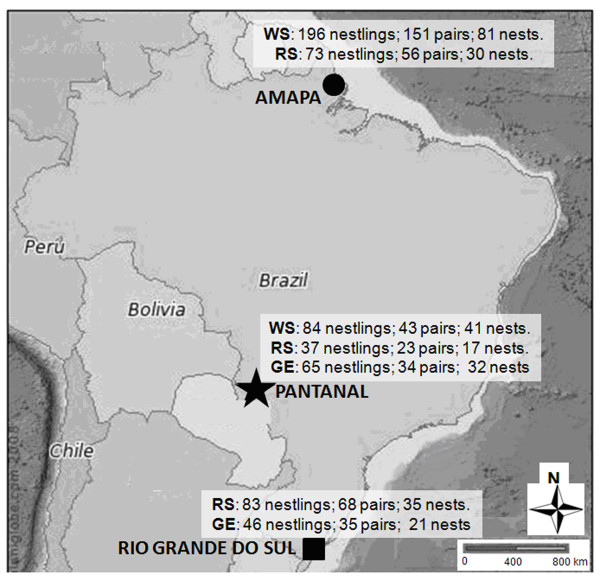
**Sampling sites and number of collected samples**. Map of Brazil showing sampling sites (Amapa: black circle, Pantanal: black star, and Rio Grande do Sul: black square) and the number of Wood Storks (WS), Roseate Spoonbills (RS) and Great Egret (GE) nestlings, nestling-pairs and nests sampled in each site.

Wood Stork (N = 280), Roseate Spoonbill (N = 193) and Great Egret (N = 111) nestlings were sampled during the 2006, 2007 and 2008 breeding seasons in colonies located in the Amapa state (AP), Pantanal (PAN) region and Rio Grande do Sul state (RS), Brazil (Figure 1; Table 1). The Pantanal is a freshwater wetland where waterbirds reproductive cycle extends for ca. five months [21]. In the Amapa state, waterbirds' colonies occur in freshwater and estuarine habitats, and breeding cycles extend for ca. six months. In Rio Grande do Sul, waterbirds' breeding colonies are established in coastal lagoons, and breeding cycles extend during ca. four months.

**Table 1:** Sampling information for Wood Stork, Roseate Spoonbill and Great Egret nestlings

Species	Breeding colony	Geographical coordinates	# nestlings	# pairs	# nests
Wood Stork, *Mycteria americana*	Fazenda Ipiranga (PAN)	16˚25' S, 56˚ 36' W	13	5	6

Wood Stork, *Mycteria americana*	Porto da Fazenda (PAN)	16˚27' S, 56˚ 07' W	25	15	12

Wood Stork, *Mycteria americana*	Tucum (PAN)	16˚26' S, 56˚ 03' W	28	14	14

Wood Stork, *Mycteria americana*	Presidente (PAN)	16°43' S, 57° 50' W	18	9	9

Wood Stork, *Mycteria americana*	Macacoari (AP)	00°27' N, 50° 40' W	109	94	42

Wood Stork, *Mycteria americana*	Fazenda Zelândia (AP)	01°09' N, 50° 23' W	12	6	6

Wood Stork, *Mycteria americana*	Fazenda Alegria (AP)	01°02' N, 50° 32' W	7	5	3

Wood Stork, *Mycteria americana*	Se Cria (AP)	01°56' N, 50° 35' W	68	46	30

Roseate Spoonbill, *Platalea ajaja*	Porto da Fazenda (PAN)	16˚27' S, 56˚07' W	15	9	7

Roseate Spoonbill, *Platalea ajaja*	Praialzinho (PAN)	16°76' S; 56°58' W	22	14	10

Roseate Spoonbill, *Platalea ajaja*	Fazenda Zelândia (AP)	01°09' N, 50°24' W	23	16	10

Roseate Spoonbill, *Platalea ajaja*	Se Cria (AP)	01°56' N, 50° 35' W	50	40	20

Roseate Spoonbill, *Platalea ajaja*	Banhado do Taim (RS)	32°29' S, 52°35' W	83	68	35

Great Egret, *Ardea alba egretta*	Porto da Fazenda (PAN)	16˚28' S, 56˚ 07' W	25	14	12

Great Egret, *Ardea alba egretta*	Praialzinho (PAN)	16°46' S, 56°35' W	30	15	15

Great Egret, *Ardea alba egretta*	Tucum (PAN)	16°26' S, 56°03' W	10	5	5

Great Egret, *Ardea alba egretta*	Barra do Ribeiro (RS)	30°16' S, 51°23' W	46	35	21

Number of Wood Stork, Roseate Spoonbill and Great Egret nestlings, pairs and nests sampled in Brazilian breeding colonies from the Amapa state (AP), the Pantanal region (PAN) and Rio Grande do Sul state (RS), analyzed for kinship in this study. Geographical coordinates are shown for each breeding colony sampled.

Nestlings were sampled at 2-3 weeks of age, when they exhibit little mobility and remain inside the nests where they were hatched [22,24,33]. At this age, they also show less susceptibility to raptors and other predators than do eggs or recently-hatched nestlings. Blood samples (N = 501) of 0.2 ml were obtained by puncture of the brachial vein of nestlings using sterile disposable syringes with 3% EDTA as anticoagulant. Growing feathers (N = 83) were plucked from Roseate Spoonbill nestlings from Rio Grande do Sul state. Both blood samples and growing feathers were stored in plastic microtubes with ethanol 96% at room temperature while in the field and then kept at -20°C until DNA isolation.

Genomic DNA was isolated from blood samples and growing feathers following a standard phenol-chloroform extraction protocol [34]. Samples were genotyped at microsatellite loci (Wood Stork: nine loci; Roseate Spoonbill: five loci; Great Egret: seven loci) through PCR amplification (see Additional file 1, Table S1 for laboratory conditions) using primers [35-37] labeled with fluorescent tags (Alpha DNA, Montreal, Canada) (Additional file 1, Table S1). Amplification reactions were carried out in a Mastercycler^® ^gradient thermal cycler (Eppendorf AG, Germany). Standard cycling parameters were: 94°C for 3 minutes, 35 cycles of 94°C for 50 seconds, locus-specific annealing temperature for 50 seconds, 72°C for 50 seconds, and a final extension of 72°C for 5 minutes. PCR products were detected on EtBr-stained agarose gels. Genotypic data were collected using a MegaBACE™1000 automatic sequencer with MegaBACE™ ET 550-R as an internal size standard (GE Healthcare, Piscataway, NY, U.S.A.). Allelic profiles of each individual at each locus were identified using MegaBACE Fragment Profiler™ software (GE Healthcare, Piscataway, NY, U.S.A.).

### Estimates of population genetic parameters

The quality of the genotypic dataset was assessed using MICRO-CHECKER[38]. Allelic diversity, observed (*H_O_*) and expected heterozygosity (*H_E_*), probability of identity (*P_I_*) [39] and probability of exclusion (*P_E_*) with one-parent known and with neither-parent known [40] were computed using GENALEX 6 [41]. Deviation from Hardy-Weinberg Equilibrium (*HWE*) was assessed using exact tests based on the Markov chain method [42], as implemented in GENEPOP 3.3 (updated from [43]) with 1,000 dememorizations, 1,000 batches and 10,000 iterations. Linkage disequilibrium was investigated for all pairs of loci using GENEPOP 3.3. Type I error rates for tests of linkage disequilibrium and departure from *HWE *expectations were corrected using the sequential Bonferroni procedure for multiple comparisons [44].

To minimise bias in subsequent relatedness analyses, the above population genetic parameters were estimated excluding from the population sample the individuals being analyzed for kinship (Wood Storks: Pantanal N = 75, Amapa N = 72; Roseate Spoonbills: Amapa N = 39, Pantanal N = 38, Rio Grande do Sul N = 74; Great Egret: Pantanal N = 67, Rio Grande do Sul N = 59).

### Performance of relatedness estimators

Genetic relatedness between the nestlings sampled inside the same nests was assessed by means of Queller and Goodnight [45] and Lynch and Ritland [46] indices (hereafter named *Q&Gr *and *L&Rr*, respectively) using KINGROUP v2.090501 [47]. To assess the performance of these relatedness estimators for our study samples, a Monte Carlo simulation approach was used to estimate sampling variance of relatedness measures for known relationship categories [48]. For each species and sample, KINGROUP was employed to generate 1,000 pairs of genotypes for unrelated (UR), half-siblings (HS) and full-siblings (FS) based on observed allele frequencies at each locus. For each estimator, the significance of potential bias for each relationship category was tested using two-tailed *t*-tests by comparing mean observed relatedness and theoretically expected relatedness values (UR: 0.0, HS: 0.25, FS: 0.5). Sampling variance of the indices was calculated as the variance of the mean relatedness estimate for each simulated data set. Critical values for significance were adjusted for multiple tests using Bonferroni correction [44] (*k *= 3, three tests per estimator). Statistical analyses were performed using BIOSTAT 3.0 [49]. To assess the power of our marker set for assigning dyads to a certain relationship category, the program IREL v1.0 [50] computed expected misclassification rates as the fraction misclassified out of 1,000 simulated pairs of each category, using the cut-off values method of [51], as described in [52]. Expected type II error rates were computed as the proportions of simulated HS and FS pairs misclassified in a lower relationship category.

### Pairwise relatedness, relationship hypotheses, significance tests and classification of nestling-pairs into a relationship category

**Figure 2:**
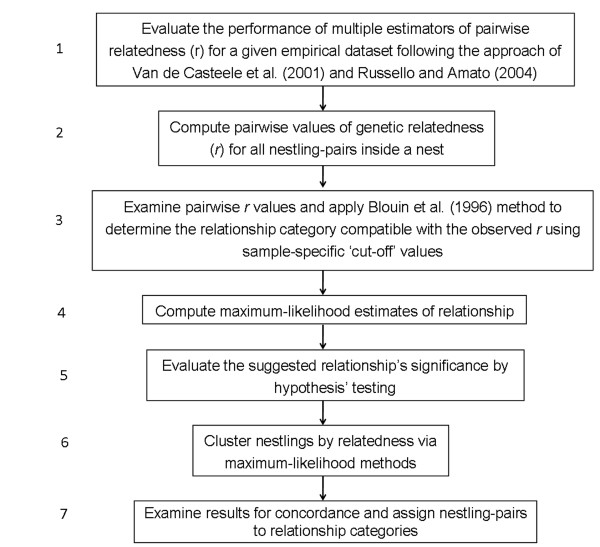
**Flow-chart outlining the methodological approach adopted for classification of nestlings-pairs**. Flow-chart outlining the seven-steps approached followed in this study to identify the most-likely kinship patterns for co-nesting nestlings of the Wood Stork, the Roseate Spoonbill and the Great Egret.

To classify nestlings into a relationship category, we followed a multi-step approach (Figure 2). First, we calculated pairwise values of genetic relatedness (*r*), with the estimator that performed best based on the simulations described in the previous section. To minimize bias in the estimation of pairwise relatedness between nestlings, we computed those values based on population allele frequencies estimated using independent population samples for each location (as described in the preceding section). Second, we applied the cut-off values method [51], based on previously calculated pairwise *r *values. Using this method, there is a greater than zero probability of misclassifying individuals if observed values of relatedness fall outside theoretically expected values [52]. To minimise this error, we calculated the cut-off values specific for our samples using the Monte Carlo simulation procedure recommended in [52]. For this, IREL[50] randomly generated 1,000 pairs of UR, HS and FS using as input data the population allele frequencies estimated for each sample, and then computed *Q&Gr *and *L&Rr *for simulated dyads. The midpoints between the means of the distributions of pairwise relatedness estimates of each simulated relationship category were taken as cut-off values [51]. The relationship category compatible with the observed *r *value was then determined for each nestling-pair. Third, we generated maximum likelihood (ML) relationship-hypotheses for each nestling-pair [7] using ML-RELATE[53]. This program compares the likelihoods of different relationship categories between two individuals, based on simulations and genotypic data, and corrects for the presence of null alleles [53]. We further assessed the significance of the relationship-hypotheses by computing the probability of the nestlings being related according to the most likely relationship versus the *a priori *expected relationship under the assumption of genetic monogamy (i. e., that two offspring in a given nest are expected to be FS). For example, if ML-RELATE indicated that a pair of nestlings was UR, this was established as the putative hypothesis (*H_P_*) and tested against an alternative hypothesis (*H_A_*) of full sibship. This procedure allowed us to maximise the power to reject the alternative hypothesis [7]. Testing of hypotheses was performed with 10,000 randomly simulated genotypes in ML-RELATE[53]. Additionally, testing of hypotheses was performed in a similar manner using KINGROUP[48], and results of these two programs were compared. Significance level of the obtained ratio in KINGROUP was calculated by simulating 1,000 pairs of individuals using the putative hypothesis settings, and the estimated allele frequencies for the population of origin of the analyzed nestlings. We rejected the alternative hypothesis if *P_α _*was *≤ *0.05. Fourth, as recommended for situations in which parental information is absent [2], we reconstructed kin groups in our samples by identifying most likely FS, HS or UR using the partial-pedigree approach of [54,55]. This method is based on a Markov Chain Monte Carlo algorithm implemented in the software PEDIGREE v2.2 (available at: http://herbinger.biology.dal.ca:5080/Pedigree). The program was run 10 times, applying the full-sib constraint (FSC) with: 500,000 iterations, a weight of one, a temperature (speed of the algorithm) of 10, and a random seed. We identified a stable solution (kin partition) after comparing the results of different runs and then used this partition as a starting point to run the program an additional 10 times with a temperature of 30 (to improve the chances of finding a better partition). We inspected the final partition to determine if nestling-pairs sampled within the same nests were recovered by the algorithm as being FS or HS. Final classification of nestlings-pairs in a relationship category was achieved if and only if all the above described methods were congruent about the relationship identified for each pair.

In addition, for all three species and regions sampled, we computed mean *r *values among nests and compared it with the average *r *observed inside nests. Also, mean *r *observed inside nests was compared to mean *r *value expected for a simulated sample of UR, FS and HS (considering our observed allele frequencies). In all cases, we applied a non-parametric test for independent samples (Mann-Whitney *U*-test) implemented in BIOSTAT 3.0 [49].

## Results

### Genetic variation parameters

There was no evidence of linkage disequilibrium for any pair of loci for any species. In the Wood Stork sample there was no evidence of departures from *HWE *for any marker and sample (Additional file 2, Table S2). MICRO-CHECKER did not detect evidence of null alleles or genotyping error due to allelic dropout or stuttering. Consequently, subsequent population genetic and relatedness analyses were run based on data at all nine loci. The number of alleles per locus ranged from 2 to 5, with a total number of 27 alleles in each sample site. Average number of alleles per locus was three across both sampling sites. Average *H_E _*was 0.38 in samples from Pantanal and 0.36 in samples from Amapa. The combined exclusion probability with one-parent known was 0.90, while the combined probability for excluding both parents was 0.95.

In the Roseate Spoonbill sample there was no evidence of departures from *HWE *for any marker and sample combination (Additional file 2, Table S2). MICRO-CHECKER did not detect evidence of null alleles or genotyping error due to allelic dropout or stuttering in Roseate Spoonbill samples. Consequently, subsequent population genetic and relatedness analyses were run based on data at all five loci. The number of alleles per locus ranged from 2 to 11, with a total number of 34 alleles in Pantanal, 34 in Amapa and 36 in Rio Grande do Sul. Average number of alleles per locus was 6.8 in Pantanal, 7.0 in Amapa and 7.2 in Rio Grande do Sul. Average *H_E _*was 0.61 in Pantanal, 0.63 in Amapa and 0.65 in Rio Grande do Sul. The combined exclusion probability with one-parent known was 0.98, while the combined probability for excluding both parents was 0.99.

In the Great Egret sample, global tests revealed significant deviations from *HWE *from both the Pantanal and Rio Grande do Sul samples (Additional file 2, Table S2). Three loci (Ah414, Ah522, Ah630) deviated from *HWE *in samples from the Pantanal region (Additional file 2, Table S2). Two loci (Ah414, Ah522) deviated from *HWE *in Rio Grande do Sul (Additional file 2, Table S2). MICRO-CHECKER analyses suggested the presence of null alleles at two loci (Ah414 and Ah522) in samples from both breeding regions, which were subsequently removed from all downstream analyses. For the remaining five loci, the number of alleles per locus ranged from 2 to 18, with a total number of 39 alleles in the global sample. Levels of genetic diversity were variable across loci and breeding regions, with average *H_E _*estimates of 0.63 and 0.53 in Pantanal and Rio Grande do Sul, respectively. The combined exclusion probability with one-parent known was 0.96, while the combined probability for excluding both parents was 0.99.

### Performance of relatedness estimators

None of the relatedness estimators deviated significantly from the theoretically expected mean relatedness values for simulated samples, considering each relationship category for any species or sample, as indicated by *P*-values of two-tailed *t*-tests (Additional file 3, Table S3). Monte Carlo simulations based on observed allele frequencies indicated that *Q&Gr *had lower sampling variances for all simulated relationships, species and samples (Additional file 3, Table S3). The *Q&Gr *had higher power in distinguishing between adjacent categories of relationship than did *L&Rr *in all species, as indicated by theoretically expected misclassification rates (Additional file 4, Table S4). For this reason, the *Q&Gr *index was chosen for subsequent analyses to assess nestlings' relationships.

### Mean relatedness among and within nests

**Figure 3:**
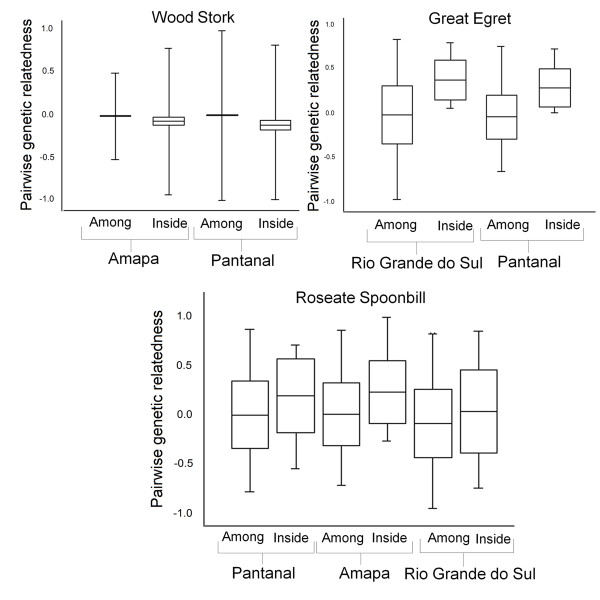
**Mean coefficient of pairwise genetic relatedness among and inside nests**. Mean coefficient of pairwise genetic relatedness among and inside nests of the Wood Stork, the Roseate Spoonbill and the Great Egret, sampled in the Amapa state, the Pantanal region and Rio Grande do Sul state, Brazil, based on microsatellite loci. Means are represented along with maximum and minimum values and ± one standard deviation.

Mean *r *observed inside Wood Stork nests (Figure 3; Additional file 5, Table S5) did not differ from mean *r *observed among nests (Pantanal: *P *= 0.109, Amapa: *P *= 0.965), nor from the expected *r *value for UR individuals (Pantanal: *P *= 0.188, Amapa: *P *= 0.075). Mean *r *observed inside Wood stork nests, however, differed from the expected *r *value for FS and also from HS in samples from both sampled regions (*P *values < 0.0001 for all comparisons).

Mean *r *observed inside Roseate Spoonbill nests (Figure 3; Additional file 5, Table S5) differed significantly from mean *r *observed among nests (Pantanal = *P *= 0.001, Amapa = *P *= 0.009, Rio Grande do Sul = *P *= 0.006). Both for Pantanal and Amapa samples, mean *r *inside Roseate Spoonbill nests differed from the *r *expected for UR individuals (Pantanal: *P *= 0.001, Amapa: *P *= 0.004), but not in Rio Grande do Sul (*P *= 0.181). Mean *r *inside Roseate Spoonbill nests did not differ from the *r *expected for HS, neither in the Pantanal (*P *= 0.519) nor in Amapa (*P *= 0.241), but it did differ from the expected for HS (*P *= 0.004) and FS in Rio Grande do Sul (*P *< 0.0001).

Mean *r *observed inside Great Egret nests (Figure 3; Additional file 5, Table S5) differed from *r *observed among nests and also from the expected *r *for UR individuals (Pantanal and Rio Grande do Sul: *P <*0.0001) and for FS (Pantanal: *P *< 0.0001, Rio Grande do Sul: *P *= 0.001). On the other hand, Great Egret samples from the Pantanal did not differ from the *r *expected for HS (*P *= 0.412), but samples from Rio Grande do Sul did (*P *= 0.004).

### Kinship patterns among co-nesting nestlings

**Figure 4:**
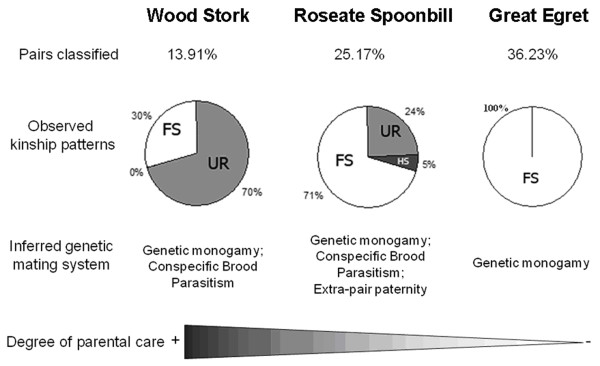
**Genetic mating system of three colonially breeding waterbirds inferred by reconstructed kinship patterns among nestmates**. Proportions of pairs classified as either full-siblings (FS), half-siblings (HS) or unrelated (UR) relative to the total number of Wood Stork, Roseate Spoonbill, and Great Egret nestlings-pairs sampled inside nests in breeding colonies from Amapa state, Pantanal Region and Rio Grande do Sul state (Brazil). Observed kinship pairs in our sample are shown for each species. Relationships were identified using the procedure outlined in figure 2 and reported in Tables S6-S12. Inferred genetic mating system and degree of parental care are also indicated.

Kinship patterns among nestmates reconstructed according to our multi-step approach allowed us to infer likely FS, HS or UR nestlings (Additional file 6, Table S6; Figure 4). We were able to identify the relationship category for 13.9% of sampled Wood Storks pairs (Figure 4), of which 88.9% were from Amapa state and 11.1% from Pantanal region. Of the pairs diagnosed in Amapa, 33.3% were FS and 66.7% were UR (Additional file 7, Tables S7), whereas all of the Wood Stork nestling-pairs from the Pantanal were UR (Additional file 8, Table S8). In terms of nests, we could classify all nestling-pairs sampled inside three Wood Stork nests from the Pantanal (7.3% of nests sampled in this region) and inside seven nests from Amapa state (11.1% of nests sampled in this region).

For Roseate Spoonbill pairs, we classified 25.2% of the analyzed nestling-pairs (Figure 4), of which 35.2% were from Amapa state, 18.9% were from the Pantanal region and 45.9% were from Rio Grande do Sul state. Full-siblings were found in Roseate Spoonbill nests (17.7% of the total of sampled pairs), including 100% of diagnosed pairs from Amapa, 57.1% of diagnosed pairs from the Pantanal, and 52.9% of diagnosed pairs from Rio Grande do Sul (Additional file 9, Table 9; Additional file 10, Table S10; Additional file 11, Table S11). To a lesser extent, HS were also detected inside Roseate Spoonbill nests from the Pantanal region (14.3% of the diagnosed pairs in this region) and in Rio Grande do Sul (5.8% of the diagnosed pairs in this region). In addition, UR nestlings were detected in the Pantanal (28.6% of the diagnosed pairs in this region) and in Rio Grande do Sul (41.3% of the diagnosed pairs in this region). In terms of nests, we classified all the nestling-pairs found within four nests from Amapa state (13.3% of nests sampled in this region), four nests from the Pantanal region (23.5% of nests sampled in this region) and six nests from Rio Grande do Sul state (17.1% of nests sampled in this region).

For Great Egret pairs, we classified 36.2% of the nestling-pairs sampled (Figure 4), of which 40% were from the Pantanal region and 60% were from Rio Grande do Sul state. All of the diagnosed nestling-pairs were identified as FS (Additional file 12, Table S12; Additional file 13, Table S13). Although some of the applied methods suggested that a low proportion of HS was present inside Great Egret nests, those results were not consistent among methods, thus, were not taken into account for our final inferences. In terms of nests, we could classify all nestling-pairs found in ten nests from the Pantanal region (31.2% of nests sampled in this region), and in 15 nests sampled in Rio Grande do Sul (71.4% of nests sampled in this region).

## Discussion

Colonially breeding waterbirds readily demonstrate ecological characteristics that have been proposed to promote both EPP and CBP, yet this group has been largely ignored to date. Breeding in colonies at high densities is one of them. In the case of EPP, high densities of breeding adults can increase the chances of encounters between fertile males and females, reducing the energetic costs for individuals searching for extra-pair mates and thus favoring increased rates of extra-pair copulations [11,56-58]. This argument has been proposed, for example, to account for high rates of extra-pair mating observed in Spanish populations of the Great Heron (*Ardea cinerea*) [59]. Moreover, high densities of nests in colonially breeding species pose significant challenges for defending against parasitism [13], and thus can favor CBP. Brazilian Wood Stork colonies in the Pantanal have up to 10,728 birds breeding together at the same time [21], a situation favourable for EPC. Recent estimates from colonies in Rio Grande do Sul reported a total of 1,508 Roseate Spoonbills [27], while colonies occurring in the Pantanal wetlands were estimated at 2,163 birds [21]. Breeding colonies from Amapa state are also dense, with nests separated, on average, only 1.5 m (S. N. Del Lama et al., unpublished data). Here, we used a multi-species and multi-step analytical approach to reconstruct genetic mating system within overlapping Brazilian breeding colonies of Wood Storks, Roseate Spoonbills and Great Egrets in order to investigate the degree to which these species may deviate from observed social monogamy and to explore the ecological and evolutionary consequences of alterative reproductive strategies within this group.

### Considerations on the methodological approach for kinship diagnosis

We inferred kinship patterns among young sharing a nest based on their genotypes, in the absence of parental information, using a combination of different methodological approaches. Our procedure considered the recommended steps to extract the most information possible when studying wild populations with limited genealogical information [7]. The different analytical approaches implemented in this study can be seen as complementary and together yielded robustness that would not have been possible with any one method alone. Some of the methods used in this study, such as the estimation of the proportion of each type of relationship category that occurs in a sample and the partition of cohorts, are among those that require few loci for obtaining accurate results [7].

In order to lend confidence to our relationship assignments, and taking into account the recommendations given in the literature [60], our methodological procedure to assign a dyad to a specific relationship category used a multi-step approach, instead of relying only on absolute pairwise values of relatedness [60]. Highest confidence relationship classifications were those for which all methods were congruent, rendering our approach conservative in order to minimize error introduced by limitations in sampling (absence of parental data) and the limited power of our set of genetic markers for distinguishing adjacent relationship categories. This criterion, although rather stringent, aimed at minimizing error in rejecting a hypothesis of genetic monogamy. The large percentage of dyads that could not be classified in any relationship category (63.7% - 85.1%, depending on the species) directly reflected the stringency of our approach. In addition, our estimates of EPP are likely greatly underestimated, as inference of this behavior requires the power to distinguish HS from UR and first-order relationships. Although the multi-step approach has these stated limitations, the fraction of nestmates effectively diagnosed provide high-confidence inferences for investigating genetic mating system of the three studied waterbird species in the absence of parental information.

### Kinship patterns between nestmates and implications for genetic mating system

Taking into account kinship patterns (i. e., whether they were likely UR, FS or HS) inferred for Wood Storks, Roseate Spoonbills and Great Egret nestmates sampled in broods from Brazilian natural breeding colonies, we were able to infer reproductive behaviors among the unobserved and unsampled parents.

For the Great Egret, our estimates provided genetic evidence for the presence of full-siblings in all sampled nests (Figure 4). These findings suggest that genetic monogamy is present in the mating system of the Great Egret, in agreement with behavioral observations reporting social monogamy for this species. However, since an important proportion of the studied dyads (63.7%) could not be classified in any relationship category, we cannot completely rule out the presence of other alternative reproductive strategies, such as extra-pair copulation, as this behaviour has been observed in previous ecological studies in this species [31,32].

Full-siblings were also found inside Wood Stork's and Roseate Spoonbill's nests, as expected under the hypothesis of genetic monogamy. However, in addition to the presence of FS, we obtained genetic evidence for the presence of unrelated nestlings inside Wood Stork (11.3% of the analyzed pairs) and Roseate Spoonbill nests (6.1% of the analyzed pairs), and the presence of half-siblings in Roseate Spoonbill broods (1.4% of the analyzed pairs). Taken together, these findings indicate that genetic monogamy is not the sole reproductive strategy exhibited by these species in the studied colonies. Specifically, CBP can explain the presence of unrelated nestmates and EPP can account for half-siblings in a brood. Moreover, our findings are consistent with an earlier study of Wood Storks that observed clutches with additional eggs after the first broods fledged [22]. Also, significant differences in sizes amongst nestlings from the same brood observed in some Wood Stork clutches from Amapa (S. N. Del Lama, unpublished data), can be interpreted as evidence of CBP as it has been in other bird species [61,62].

Behavioural observations support CBP in other colonial Ciconiiformes and Pelecaniiformes including: *Ardea purpurea, Ardeola ralloides, Egretta rufescens *[63]*, Ciconia maguari *[14], *Eudocimus albus *[14,64,65] and *Eudocimus ruber *[66-68], although no genetic studies have been conducted in these species to date. Furthermore, behavioral observations also support the occurrence of EPC in natural populations of *Ardea alba *[31,32] and in other Ciconiiformes and Pelecaniiformes, including: *Platalea leucorodia *[69], *Ardea cinerea *[9,59], *Eudocimus albus *[70,71], *Bulbucus ibis *[72-74], *Eudocimus ruber *[67] and *Egretta eulophotes *[75]. Additional behavioral evidence of EPC and adult care of infant nestlings other than their own is available for captive populations of the Roseate Spoonbill [76]. Those *ex-situ *observations were further supported by genetic parentage analyses which confirmed both reproductive strategies [77].

### Ecological and evolutionary consequences of alternative reproductive strategies in waterbirds

Findings from this study are consistent with the observation that much variation occurs in genetic mating system between closely related species that apparently have very similar biology [78,1]. According to our results, while the Wood Stork mostly showed evidence of CBP, the Roseate Spoonbill presented CBP and EPP, and the Great Egret appears to conform to a monogamous genetic mating system. Both alternative reproductive strategies revealed in this study, CBP and EPP, have been demonstrated by molecular methods to be frequent phenomena in avian populations [1,79-81]. We can only speculate as for the possible causes of the differences in EPP and CBP levels observed among the three waterbird species analyzed in this study. We suggest that differences in life-history traits amongst the studied species, such as in the amount of parental care, can play a role in shaping the observed differences in levels of CBP. An advantage of CBP for species that have relatively high reproductive costs associated with parental care, such as the Wood Stork and the Roseate Spoonbill, is that this behavior provides an opportunity to enhance the reproductive success of parasitic females [79,82-84]. By ensuring that its offspring would receive appropriate parental care through development, a female can increases its fitness. In addition, by laying eggs in nests of other conspecifics, a female can increase the chances of its offspring being adequately protected from predation [85]. Limited ecological observations for the Roseate Spoonbill indicated that 54% of nests failed during the 2008/9 breeding season in Rio Grande do Sul. From those failures, 73% were due to egg predation and 25% due to problems in nestlings' development [27]. For the Wood Stork, limited ecological observations have also indicated that predation pressure can be high. In Pantanal breeding colonies of this species, Bouton [18] identified up to 16 different animal species that predated on adult birds and their nestlings. Thus, taking into consideration both the relatively high pressures imposed by predation and nest-failure, CBP can be a good alternative reproductive tactic in the Wood Stork and in the Roseate Spoonbill. On the other hand, Great Egrets may not be as prone to CBP as an alternative reproductive strategy, given lower predation rates [33] and reduced commitment to parental care given the semi-altricial nature of their young.

Conspecific brood parasitism has been proposed to be frequent in colonially nesting species [12,13,86] because colonial breeders would benefit from the close proximity and higher availability of potential host nests of other reproductive conspecifics for egg laying [62,79,87,88]. Colonial reproductive behaviour also increases the chances of encounters between fertile males and females and reduces the energetic costs for individuals searching for extra-pair mates, both of which may contribute to elevated rates of extra-pair copulations [11,56-58].

Although the aspects of waterbirds' reproductive tactics inferred from our kinship analyses are noteworthy, additional studies with a larger set of markers would help to more accurately estimate the frequency of CBP and EPP in these species. Coupled with enhanced sampling of adults, this would allow for more direct investigations of within and among nest relatedness as well as providing an explicit test of our analytical approach. Lastly, the addition of ecological data would complement the results from this genetic study for more comprehensively characterizing mating systems in these Neotropical waterbirds.

## Conclusions

In conclusion, we have shown that alternative reproductive strategies are likely present in natural populations of Wood Storks and Roseate Spoonbills, whereas Great Egrets did not deviate from a hypothesis of genetic monogamy. Our results can contribute to guide future ecological studies and stimulate increased sampling efforts, including the exploration of alternative sampling strategies. Overall, the methodological approach implemented here may have broad application for estimating kinship patterns in systems with limited genealogical information and/or in studying similarly challenging organisms in which obtaining biological materials from complete families is problematic.

## Authors' contributions

CIM participated in the design of the study, sample collection, carried out the molecular genetic analyses, performed the statistical analysis, interpreted results and drafted the manuscript; SNDL participated in sample collection and study design, coordinated the study, contributed to interpretation of results and helped to draft the manuscript; MAR contributed to data analyses' design, interpretation of results and helped to draft the manuscript; PFMG produced part of the Wood Stork molecular data. All authors have been involved in drafting the manuscript and revising it critically for important intellectual content. All authors read and approved the final manuscript.

## Authors' information

This manuscript is part of CIM doctoral thesis. CIM is now a postdoctoral researcher at the Department of Genetics and Evolution (Universidade Federal de São Carlos) investigating on the distribution of genetic diversity at different hierarchical levels in animal species, with focus on conservation. MAR is an Assistant Professor at The University of British Columbia-Okanagan Campus with research interests in vertebrate conservation genetics, molecular ecology and population genomics. PFMG was an undergraduate student at Universidade Federal de São Carlos at the time of this study. SNDL is a Professor at Universidade Federal de São Carlos with research interest in conservation genetics and ecology of waterbird populations.

## Supplementary Material

Additional file 1 **Table S1 - Excel spreadsheet**. Laboratory conditions for polymerase chain reaction (PCR) of microsatellite loci amplified in the Wood Stork, the Roseate Spoonbill and the Great Egret. Wood Stork and Roseate Spoonbill microsatellites were amplified using 0.5 U *Taq *DNA polymerase (Fermentas Inc, Glen Burnie, MD, U. S. A.); Great Egret microsatellites were amplified using 1U *Taq *DNA polymerase (Biotools B&M Labs, S.A., Madrid, Spain). Click here for file

Additional file 2 **Table S2 - Excel spreadsheet**. Estimates of population-genetic parameters of microsatellite diversity for Roseate Spoonbill, Wood Stork and Great Egret samples from Amapa State, Pantanal region and Rio Grande do Sul State, Brazil. N: number of individuals analyzed, *A*: number of alleles, *H_O_: *observed heterozygosity, *H_E_: *expected heterozygosity, *P_EHW_: *probability of the exact test for deviation from Hardy-Weinberg Equilibrium, *P_E_: *single-locus probability of exclusion (Waits et al. 2001), and *PI*: probability of identity (Jamieson & Taylor 1997) for increasing locus-combinations. Click here for file

Additional file 3 **Table S3 - Excel spreadsheet**. Genetic relatedness ± standard error of Queller & Goodnight's index (1989) (*Q&Gr*) and of Lynch & Ritland's index (1999) (*L&Rr*) for 1.000 simulated pairs of unrelated (UR), half-sibs (HS) and full-sibs (FS), based on allelic frequencies observed in Roseate Spoonbills, Wood Storks and Great Egrets sampled in Amapa state, Pantanal region and Rio Grande do Sul state, Brazil. In parentheses, variances for each relationship category; lower variances are shown in bold. *P*-values of two-tailed *t*-tests for significance for differences between simulated and theoretically expected relatedness values (UR: 0; FS: 0.50; HS: 0.25) are shown in italics [(t0.05(2).999 = 1.962; critical *P-*value after Bonferroni correction = 0.0166]. Click here for file

Additional file 4 **Table S4 - Excel spreadsheet**. Misclassification rates expected for Roseate Spoonbill, Wood Stork and Great Egret nestling-pairs (Queller & Goodnight's 1989 index: *Q&Gr; *and Lynch & Ritland's 1999 index: *L&Rr*), estimated as the proportion misclassified out of 1.000 simulated pairs of unrelated (UR), half-siblings (HS) and full-siblings (FS), based on allele frequencies observed in samples from Amapa state, Pantanal region and Rio Grande do Sul state (Brazil) (simulations performed as in Russello & Amato, 2004). Click here for file

Additional file 5 **Table S5 - Excel spreadsheet**. Mean value of pairwise relatedness (*r*) (in italics) ± standard deviation (S.D.) estimated among and inside nests for each species and sample site. Click here for file

Additional file 6 **Table S16 - Excel spreadsheet**. Number of dyads examined, number of dyads categorized and number of dyads in each one of the relationship categories, for each species: full-siblings (FS), half-siblings (HS) or unrelated (UR). Click here for file

Additional file 7 **Table S7 - Excel spreadsheet**. Kinship patterns for Wood Stork (*Mycteria americana*) nestling-pairs sampled inside nests in breeding colonies from Amapa state, Brazil. For each pair, pairwise relatedness value (Queller & Goodnight 1989 index; *Q&Gr*), most likely relationship category (ML-R, as indicated by the maximum likelihood method in the program ML-Relate, Kalinowski et al. 2006), probability value (*P*) of hypothesis testing to establish the significance of the ML-R category (*H_P_*: putative hypothesis; *H_A_*: alternative hypothesis: a *P *value < 0.005 indicates that *H_P _*is more in agreement with the genetic data than the H*_A_*), and the *score *value for kinship reconstruction in PEDIGREE (Herbinger, 2006) are shown. All estimates were computed based on allele frequencies observed in nine microsatellite loci. UR: unrelated; FS: full-siblings; HS: half-siblings. Click here for file

Additional file 8 **Table S8 - Excel spreadsheet**. Kinship patterns for Wood Stork (*Mycteria americana*) nestling-pairs sampled inside nests in breeding colonies from Pantanal region, Brazil. For each pair, pairwise relatedness value (Queller & Goodnight 1989 index; *Q&Gr*), most likely relationship category (ML-R, as indicated by the maximum likelihood method in the program ML-Relate, Kalinowski et al. 2006), probability value (*P*) of hypothesis testing to establish the significance of the ML-R category (*H_P_*: putative hypothesis; *H_A_*: alternative hypothesis: a *P *value < 0.005 indicates that *H_P _*is more in agreement with the genetic data than the H*_A_*), and the *score *value for kinship reconstruction in PEDIGREE (Herbinger, 2006) are shown. All estimates were computed based on allele frequencies observed in nine microsatellite loci. UR: unrelated; FS: full-siblings; HS: half-siblings; Relat.: relationship identified. Click here for file

Additional file 9 **Table S9 - Excel spreadsheet**. Kinship patterns for Roseate Spoonbill (*Platalea ajaja*) nestling-pairs sampled inside nests in breeding colonies from Amapa State, Brazil. For each pair, pairwise relatedness value (Queller & Goodnight 1989 index; *Q&Gr*), most likely relationship category (ML-R, as indicated by the maximum likelihood method in the program ML-Relate, Kalinowski et al. 2006), probability value (*P*) of hypothesis testing to establish the significance of the ML-R category (*H_P_*: putative hypothesis; *H_A_*: alternative hypothesis: a *P *value < 0.005 indicates that *H_P _*is more in agreement with the genetic data than the H*_A_*), and the *score *value for kinship reconstruction in PEDIGREE (Herbinger, 2006) are shown. All estimates were computed based on allele frequencies observed in five microsatellite loci. UR: unrelated; FS: full-siblings; HS: half-siblings. Click here for file

Additional file 10 **Table S10 - Excel spreadsheet**. Kinship patterns for Roseate Spoonbill (*Platalea ajaja*) nestling-pairs sampled inside nests in breeding colonies from Pantanal region, Brazil. For each pair, pairwise relatedness value (Queller & Goodnight 1989 index; *Q&Gr*), most likely relationship category (ML-R, as indicated by the maximum likelihood method in the program ML-Relate, Kalinowski et al. 2006), probability value (*P*) of hypothesis testing to establish the significance of the ML-R category (*H_P_*: putative hypothesis; *H_A_*: alternative hypothesis: a *P *value < 0.005 indicates that *H_P _*is more in agreement with the genetic data than the H*_A_*), and the *score *value for kinship reconstruction in PEDIGREE(Herbinger, 2006) are shown. All estimates were computed based on allele frequencies observed in five microsatellite loci. UR: unrelated; FS: full-siblings; HS: half-siblings. Click here for file

Additional file 11 **Table S11 - Excel spreadsheet**. Kinship patterns for Roseate Spoonbill (*Platalea ajaja*) nestling-pairs sampled inside nests in breeding colonies from Rio Grande do Sul, Brazil. For each pair, pairwise relatedness value (Queller & Goodnight 1989 index; *Q&Gr*), most likely relationship category (ML-R, as indicated by the maximum likelihood method in the program ML-Relate, Kalinowski et al. 2006), probability value (*P*) of hypothesis testing to establish the significance of the ML-R category (*H_P_*: putative hypothesis; *H_A_*: alternative hypothesis: a *P *value < 0.005 indicates that *H_P _*is more in agreement with the genetic data than the H*_A_*), and the *score *value for kinship reconstruction in PEDIGREE (Herbinger, 2006) are shown. All estimates were computed based on allele frequencies observed in five microsatellite loci. UR: unrelated; FS: full-siblings; HS: half-siblings. Click here for file

Additional file 12 **Table S12 - Excel spreadsheet**. Kinship patterns for Great Egret (*Ardea alba egretta*) nestling-pairs sampled inside nests in breeding colonies from Pantanal region, Brazil. For each pair, pairwise relatedness value (Queller & Goodnight 1989 index; *Q&Gr*), most likely relationship category (ML-R, as indicated by the maximum likelihood method in the program ML-Relate, Kalinowski et al. 2006), probability value (*P*) of hypothesis testing to establish the significance of the ML-R category (*H_P_*: putative hypothesis; *H_A_*: alternative hypothesis: a *P *value < 0.005 indicates that *H_P _*is more in agreement with the genetic data than the H*_A_*), and the *score *value for kinship reconstruction in PEDIGREE (Herbinger, 2006) are shown. All estimates were computed based on allele frequencies observed in five microsatellite loci. UR: unrelated; FS: full-siblings; HS: half-siblings. Click here for file

Additional file 13 **Table S13 - Excel spreadsheet**. Kinship patterns for Great Egret (*Ardea alba egretta*) nestling-pairs sampled inside nests in breeding colonies from Rio Grande do Sul, Brazil. For each pair, pairwise relatedness value (Queller & Goodnight 1989 index; *Q&Gr*), most likely relationship category (ML-R, as indicated by the maximum likelihood method in the program ML-Relate, Kalinowski et al. 2006), probability value (*P*) of hypothesis testing to establish the significance of the ML-R category (*H_P_*: putative hypothesis; *H_A_*: alternative hypothesis: a *P *value < 0.005 indicates that *H_P _*is more in agreement with the genetic data than the H*_A_*), and the *score *value for kinship reconstruction in PEDIGREE (Herbinger, 2006) are shown. All estimates were computed based on allele frequencies observed in five microsatellite loci. UR: unrelated; FS: full-siblings; HS: half-siblings. Click here for file
